# Chemical Constitute and Larvicidal Activity of Fractions of *Ajuga chamaecistus tomentella* Plant against Malaria Vector *Anopheles stephensi*

**Published:** 2017-03-14

**Authors:** Mahnaz Khanavi, Behnaz Najafi, Seyede Nargess Sadati, Mohammad Reza Abai, Hassan Vatandoost

**Affiliations:** 1 Department of Pharmacognosy and Persian Medicine and Pharmacy Research Center, Faculty of Pharmacy, Tehran University of Medical Sciences, Tehran, Iran; 2 Department of Traditional Pharmacy, School of Traditional Iranian Medicine, Tehran University of Medical Sciences, Tehran, Iran; 3 Department of Medical Entomology and Vector Control, School of Public Health, Tehran University of Medical Sciences, Tehran, Iran

**Keywords:** *Ajuga chamaecistus* subspecies *tomentella*, Larvicidal, *Anopheles stephensi*, Phytoecdysteroid

## Abstract

**Backgrand:**

The genus *Ajuga*, belongs to Lamiaceae family, is one of the exclusive subspecies in the flora of Iran. The plants of this genus are used traditionally for treatment of joints pain, gout, jaundice, and as insecticide.

**Methods:**

larvicidal activity of methanol 80% extract and partition fractions of hexane, chloroform, and ethyl acetate obtained from aerial parts of *Ajuga chamaecistus* subspecies *tomentella* against malaria vector *An. stephensi* was evaluated. Phytochemical study of active fraction was analyzed using column chromatography and spectroscopy.

**Results:**

According to the results, among different fractions, hexane fraction has the most larvicidal activity with mortality rate of 100% in concentration of 102 ppm and LC_50_ of 95.66ppm. The structure of compound 1, main phytoecdysteroid compound separated from hexane fraction, was determined to be ajugalide-E.

**Conclusion:**

The results suggested that the hexane fraction of *Ajuga chamaecistus* subsp *tomentella* could be used as a natural and biodegradable insecticide.

## Introduction

Mosquitoes are the main vector in transmission of malaria that is still a major endemic disease in foci located in south and southeast of Iran. These areas include the provinces of Sistan and Baluchistan, Hormozgan and Kerman. Among all species of *Anopheles* recognized in Iran, 8 of them are considered as malaria vectors including: *An. culicifacies*, *An. stephensi*, *An. dthali*, *An. fluviatilis*, *An. superpictus*, *An. pulcherrimus*, *An. sacharovi*, and *An. Maculipenni* (Doosti et al. 2006). Malaria control is an important goal in developing tropical countries. Mosquito controls, using synthetic chemical insecticides have adverse effects on the environment and also cause growing of insecticide resistance in arthropods (Edrissian 2006, Khanavi et al. 2011). Plants, rich in bioactive phytochemicals, have been investigated as a source of alternative agents for control of mosquitoes. Several extract and essential oil of certain plants showed toxic effect against some public health pests (Hadjiakhoondi et al. 2003, Vatandoost et al. 2004, Hadjiakhoondi et al. 2006, Govindarajan et al. 2011, Sedaghat et al. 2011).

The genus *Ajuga* (Lamiaceae) with common name of Bugle is found in China, Korea, Japan and throughout Europe. Five species of this annual and perennial genus are found in Iran. *Ajuga chamaecistus* contains several exclusive subspecies, including *A. chamaecistus* subspecies *tomentella* (Mozaffarian et al. 2007). Some species belonging to this genus are used in traditional medicine of different countries in the world. Moreover in Iranian traditional medicine, the genus *Ajuga* (*Kamaphytus*, *Jaadeh*) has been used for treatment of joint pain, gout, and jaundice and as insecticide (Naghibi et al. 2005, Jorjani 2012). Several biological studies have been performed on many species of this genus which have confirmed their ethno pharmacological properties such as hypoglycemic (Hilaly et al. 2002), anti-inflammatory (Gautam et al. 2011), anabolic, analgesic, anti-arthritis, antipyretic, hepatoprotective, antibacterial, antifungal, antioxidant, cardiotonic (Israili et al. 2009), treatment of joint diseases (Ono et al. 2009), and their application as anti-malarial (Kuria et al. 2001). Antifeedant activity of *Ajuga iva* and *Ajuga pseudoiva* extract and their active compounds against larvae of *Sodoptera littoralis* (Egyptian cotton leafworm) have been shown in some literatures (Bondì et al. 2000, Ben Jannet 2000, Ben Jannet et al. 2001).

Prior to this study, some phytochemicals such as 20-hydroxyecdysone, cyasterone, ajugalactone, makisterone A, and 24-dehydroprecyasterone (phytoecdysteroids), 8-acetylharpagide (iridoid), *cis*- and *trans*-melilotoside, lavandulifolioside, leonoside B, and martynoside (phenylethanoid glycosides), were identified from diethyl ether and n-butanolic fractions of *Ajuga chamaecistus* ssp. *tomentella*. Cytotoxicity evaluation of some fractions of this plant showed the cytotoxicity of hexane fraction against normal and cancer cell lines (Sadati et al. 2012 a, b).

The aim of this study was to evaluate larvicidal activity of a methanol 80% extract and partition fractions of hexane, chloroform, and ethyl acetate obtained from aerial parts of *Ajuga chamaecistus* subsp *tomentella* against malaria vector *An. stephensi*. Furthermore, we performed a phytochemical investigation on the hexane fraction to identify the main components.

## Materials and Methods

### Plant material

Aerial parts of *Ajuga chamaecistus* ssp *tomentella* were collected from Tehran, Iran, in June 2008 and verified by Prof GH Amin. A voucher specimen (THE-6697) has been deposited in the herbarium of the Department of Pharmacognosy, Faculty of Pharmacy, Tehran University of Medical sciences, Tehran, Iran.

### Preparation of total extract and fractions

The air-dried and ground plants of *A. chamaecistus* ssp *tomentella* (250g) were extracted with methanol 80% at room temperature and concentrated under reduced pressure to give a dark brown extract. The extract (30g) was loaded on Silica gel (mesh 230–400) column and eluted with 250mL of hexane, chloroform, ethyl acetate and methanol 80%, separately. Finally the whole collected fractions were dried by the rotary evaporator and then by a vacuum oven.

### Preparing stock solutions

Primary tests were performed to determine the concentration of stock solutions. According to the results, the concentration of 320 ppm, 160ppm, 2560ppm, was determined for total extract, hexane fraction and methanol fraction respectively. Next, the stock solutions were serially diluted to obtain logarithmic concentrations of solution for the test. For better solubility, DMSO was used as the solvent for hexane fraction and methanol for total extract and methanol fraction. These two solvent are completely safe for larvae as it proved in controls.

### Larvicidal assays

Larvicidal activity assays of *Ajuga chamaecistus* subsp *tomentella* on the larvae of *An. stephensi* were performed on the basis of WHO protocol. The insectary condition was 30±1 °C, 60±5% relative humidity and 10:14, dark: light periods. The mosquitoes were collected from malarious areas of Iran and then maintained at the Department of Medical Entomology and Vector Control, School of Public health, Tehran University Medical Sciences. 1ml of prepared solution was mixed thoroughly with 224ml water in 400ml glass beakers. 25ml water containing 25 late instar larvae was slowly added. Four replicates maintained for each concentration. A control was set for all series of tests (1 ml of solvent was used instead of 1 ml of extract). Mortality was counted after 24 h recovery period. LC_50_ (lethal concentration to cause 50% mortality in the population) and LC_90_ (lethal concentration to cause 90% mortality in the population) were determined by the use of regression line employed by Finney (Finney 1971, WHO 2014).

### Chromatography

The hexane fraction (4g) was selected for phytochemical studies. Thus, it was chromatographed on silica gel (mesh 230–400) eluting with a gradient of chloroform-methanol (9–1) to 100% methanol to afford 4 fractions. Fraction 2 was purified with a few amount of methanol and compound 1 (36.5 mg) was obtained.

### General experimental procedures

^1^H- and ^13^C-NMR were measured in CDCL_3_ solution on a Bruker Avance spectrometer (500MHz, TMS as internal standard) for compounds **1**. FT-IR spectra determined using a Nicolet 550-A spectrometer (KBr disks). Column chromatography was acchived on Silica gel 60 (230–400 mesh, Merck) and RP-18 (Merck).

### Spectroscopic data

Ajugalide-E (**1**): White amorphous powder, FT-IR ν_max_ cm^−1^: 3237, 2953, 1735, 1689, 1248, 1029. **^1^**H and ^13^C NMR (CDCL_3_), see Table 2.

## Results

**Fig. 1.**
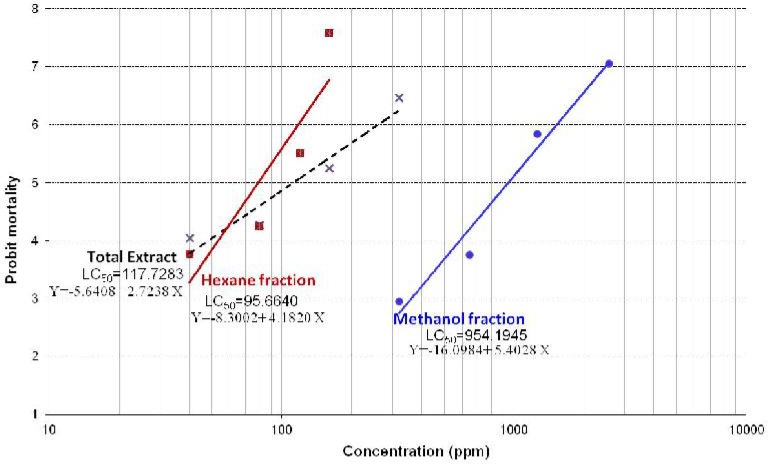
Comparison of regression lines and equations of and total extract two fractions of *Ajuga chamaepitys* subspecies *tomentella* against larvae of *Anopheles stephensi*

Larvicidal activity of the methanol 80% extract and partition fractions of hexane, chloroform, and ethyl acetate obtained from aerial parts of *Ajuga chamaecistus tomentella* against malaria vector *An. stephensi* was examined in different concentrations. According to the results presented in Fig. 1, the regression line was plotted for each extract and LC_50_ was calculated. Among the extracts, hexane fraction showed the most larvicidal effect with LC_50_ value of 95.66 ppm. LC_50_ for total extract and methanolic 80% fraction was 117.72 and 954.19ppm respectively. Also, other statistical parameters were calculated (Table 1, Fig. 1).

**Table 1.** Lethal concentrations and other associated statistic of bioassay tests of some

**Extracts**	**A**	**b ± SE**	**LC_50_ (ppm) ± 95%C.L.**	**LC_90_ (ppm) ± 95%C.L.**	**λ^2^ (heterogeneity)**	**λ^2^ table (df)**	**p-Value**
**Total extract**	−5.6408	2.7238 ± 0.252	103.6986 **117.7283** 133.4075	285.1837 309.0209 455.8304	10.704 *	13.345 (2)	0.01
**Hexane fraction**	−8.3002	4.1820 ± 0.199	88.5023 **95.6640** 105.6703	170.0557 195.5219 236.1008	16.614 *	13.345 (2)	0.01
**Methanolic 80% fraction**	−16.0984	5.4028 ± 0.643	848.9428 **954.1945** 1071.6982	1420.8157 1647.5819 2045.8799	5.002 *	9.210 (2)	0.01

* No heterogeneity

**Fig. 2.**
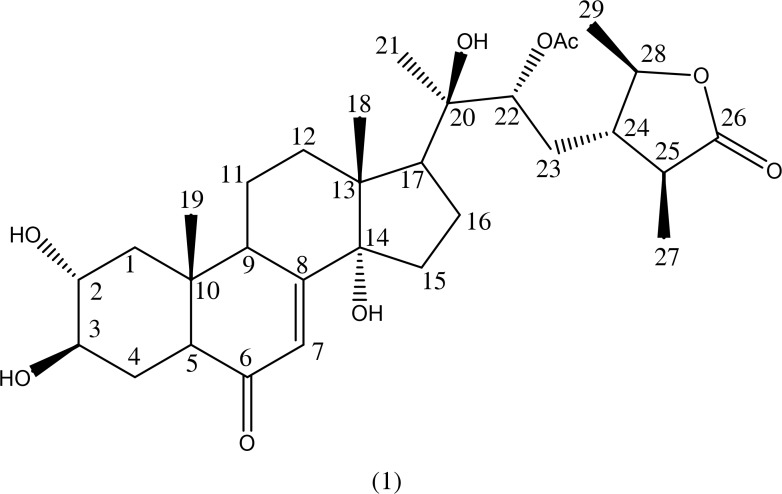
Chemical structure of Ajugalide-E (1), isolated from hexane fraction of *Ajuga chamaepitys* subspecies *tomentella*

Isolated compounds 1, 2 from the hexane fraction of total methanolic extract of aerial parts of *Ajuga chamaecistus* ssp *tomentella* were identified by comparison of their NMR (^1^H-, ^13^C-NMR) data with those reported in the literature. ^1^H and ^13^C NMR data of these compounds run in CDCL_3_ reported for the first time. δ_H_ and δ_C_ (ppm) of compound 1 was noted in Table 2. The isolated compound 1 (Fig. 2) were identified as ecdysteroids, ajugalide-E in comparison with the literature (Chan et al. 2005).

**Table 2.** ^1^H NMR and ^13^C NMR data of Ajugalide E (500 MHz in CDCL_3_)

**position**	**δ_H_**	**δ_C_**
**2**	1.98 (td)	
**3**	2.80(dd)	55.71
**7**	5.25(brs)	122.9
**8**	-	144.0
**13**	-	47.92
**14**	-	81.34
**18**	0.88(s)	17.6
**19**	0.72(s)	18.5
**21**	1.10(s)	20.7
**22**	4.47(t)	75.95
**26**	-	184.7
**27**	0.91(d)	15.8
**29**	0.84(d)	
**OAC**	2.02(s)	171.4

## Discussion

The larvicidal activity of several plant extracts and phytochemicals against mosquito larvae has been established. In a study reported by Sharma, et al. (2004) petroleum-ether extract of *Ajuga remota* was the most effective extract with LC_50_ values of 0.033% after 24 hours and 0.029% after 48 hours of treatment against the larvae of *An. stephensi* (Sharma et al. 2004). Govindarajan et al. (2011) have reported that benzene extract of *E. coronaria* showed the highest larvicidal effect on the larvae of *An. stephensi*, *Ae. aegypti*, and *Cx. quinquefasciatus* with the LC_50_ and LC_90_ values were 79.08, 89.59, and 96.15 ppm and 150.47, 166.04, and 174.10 ppm, respectively (Edrissian GhH 2006). Manjari et al. (2014) indicated that acetone leaf extracts of *Clausena dentata* showed the larval mortality against the fourth instar larvae of *An. stephensi*, *Cx. quinquefasciatus*, and *Ae. aegypti* (Diptera: Culicidae). *Culex quinquefasciatus* (LC_50=_ 0.150278mg/ml, LC_90_= 7.302613mg/ml), *A. aegypti* (LC_50_= 0.169495 mg/ml, LC_90_= 1.10034mg/ml), and *An. stephensi* (LC_50_=0.045684 mg/ml, LC_90=_ 0.045684mg/ml) (Manjari et al. 2014). In this study hexane fraction of methanolic extract of *Ajuga chamaecistus* ssp *tomentella* showed the most larvicida activity against *An. stephensi* larvae. In order to find the active ingredient of effective fraction, hexane fraction was chromatographed on silica gel and RP-18 resulted in isolation and identification of two phytoecdysteroid epimers, Ajugalide-E and 22-acetylcyasterone. In plants of genus *Ajuga*, a variety of phytoecdysteroids have been identified among them, 20-hydroxyecdysone (β-ecdysone) and cyasterone are the most abundant (Ramazanov et al. 2005). This group of natural products produces a wide range of pharmacological activities in mammals including adaptogenic, anabolic, antidiabetic, hepatoprotective, immunoprotective, wound-healing, antioxidant and free radical scavenging activities (Sadati et al. 2012 b). Phytoecdysteroids, present in many plants, are analogues of insect moulting hormone (ecdysteroids) that control insect growth, development, and reproduction (Rharrabe et al. 2010). Toxicity of some ecdysone agonists on larvae of three mosquito species, *Ae. aegypti*, *An. gombiae*, and *Cx. quinquefasciatus* have been stablished (Beckage et al. 2004). Nyamoita et al. (2013) investigated that four phytoecdysteroids isolated from aceton extract of *Vitex Schiliebenii* showed potent toxic effect against larvae of *An. gambiae* (Nyamoita et al. 2013). According to the result of this study the hexane fraction of total methanolic extract of the aerial parts of *Ajuga chamaecistus* ssp. *tomentella* exhibited the most toxicity on *An. stephensi* larvae than the other fractions. In previous study two major ecdysteroid in addition to three minor ones identified from this plant (Sadati et al. 2012b). Thin layer chromatography of the hexane fraction showed two main compounds and analyzing of this fraction resulted in isolation and identification of a phytoecdysteroid, ajugalide-E. Comparison of our result with other reports indicated that the hexane extract of the studied plant was effective to control *An. stephensi*. Further analysis of hexane fraction to isolate the active component for larval control resulted in identifying the major phytoecdysteroid compound. Based on our results it can be concluded that phytoecdysteroids are interesting molecules that can be considered as natural and biodegradable insecticide.

## Conclusion

More investigation is required to assess the larvicidal activity of the product at the field situation.
